# Recent advances in traumatic brain injury

**DOI:** 10.1007/s00415-019-09541-4

**Published:** 2019-09-28

**Authors:** Abdelhakim Khellaf, Danyal Zaman Khan, Adel Helmy

**Affiliations:** 1 Division of Neurosurgery, Department of Clinical Neurosciences, University of Cambridge, Addenbrooke’s Hospital, Box 167, Hills Road, Cambridge, CB2 0QQ UK; 2 Faculty of Medicine, McGill University, Montreal, Canada

**Keywords:** Traumatic brain injury, TBI, Therapy, Monitoring, Critical care, Neuroprotection, Neurosurgery

## Abstract

Traumatic brain injury (TBI) is the most common cause of death and disability in those aged under 40 years in the UK. Higher rates of morbidity and mortality are seen in low-income and middle-income countries making it a global health challenge. There has been a secular trend towards reduced incidence of severe TBI in the first world, driven by public health interventions such as seatbelt legislation, helmet use, and workplace health and safety regulations. This has paralleled improved outcomes following TBI delivered in a large part by the widespread establishment of specialised neurointensive care. This update will focus on three key areas of advances in TBI management and research in moderate and severe TBI: refining neurointensive care protocolized therapies, the recent evidence base for decompressive craniectomy and novel pharmacological therapies. In each section, we review the developing evidence base as well as exploring future trajectories of TBI research.

## Introduction

Traumatic brain injury (TBI) can be defined as the disruption in brain function, or other evidence of brain pathology, caused by an external physical force [1]. The yearly incidence of TBI is estimated at 50 million cases worldwide; thus, approximately half of the global population will have an episode of TBI in their life [2]. In the UK, it is the most common cause of death and disability in those aged under 40 years [3]. Moreover, even higher rates of morbidity and mortality are seen in low-income and middle-income countries [2]. Yearly, TBI costs the global economy approximately 400 billion US dollars, representing 0.5% of the gross world product [2].

TBI is a heterogeneous entity, reflecting several underlying macroscopic modes of injury (e.g., extrinsic compression from mass lesion, contusion, diffuse axonal injury [DAI]) as well as a range of mechanisms by which neuronal injury can be inflicted (e.g., ‘classical’ ischaemia, apoptosis, mitochondrial dysfunction, cortical spreading depression [CSD], and microvascular thrombosis) in differing proportions with resultant varying clinical courses [4, 5]. Practically, the clinical severity of TBI has long been stratified according to post-resuscitation Glasgow Coma Scale scores into mild (GCS 14–15), moderate (9–13), and severe (3–8) [6, 7]. Severe TBI has mortality rates of 30–40% and can cause significant physical, psychosocial, and social deficits in up to 60% of cases [8, 9].

There has been a secular trend towards reduced incidence of severe TBI in the first world, driven by public health interventions such as seatbelt legislation, helmet use, and workplace health and safety regulations. This has paralleled improved outcomes following TBI delivered in large part by the widespread establishment of specialised neurointensive care [10].

This update will focus on three key areas of advances in TBI management and research in moderate and severe TBI: refining neurointensive care protocolized therapies, establishing the evidence base for decompressive craniectomy, and developing novel pharmacological therapies.

## Refining neurocritical care and monitoring

The concept of primary and secondary injuries arose more than 25 years ago from a recognition that alongside the initial insult at the time of trauma, additional insults such as hypotension and hypoxia could supervene and exacerbate brain injury [11]. This simple concept has shaped TBI management in two ways: first, pre-hospital care protocols that ensure airway protection, systemic oxygenation, and adequate systemic perfusion and, second the use of monitoring and goal-directed therapy of neuronal physiology in the neurosciences critical care unit.

### Intracranial pressure (ICP) monitoring

**Fig. 1:**
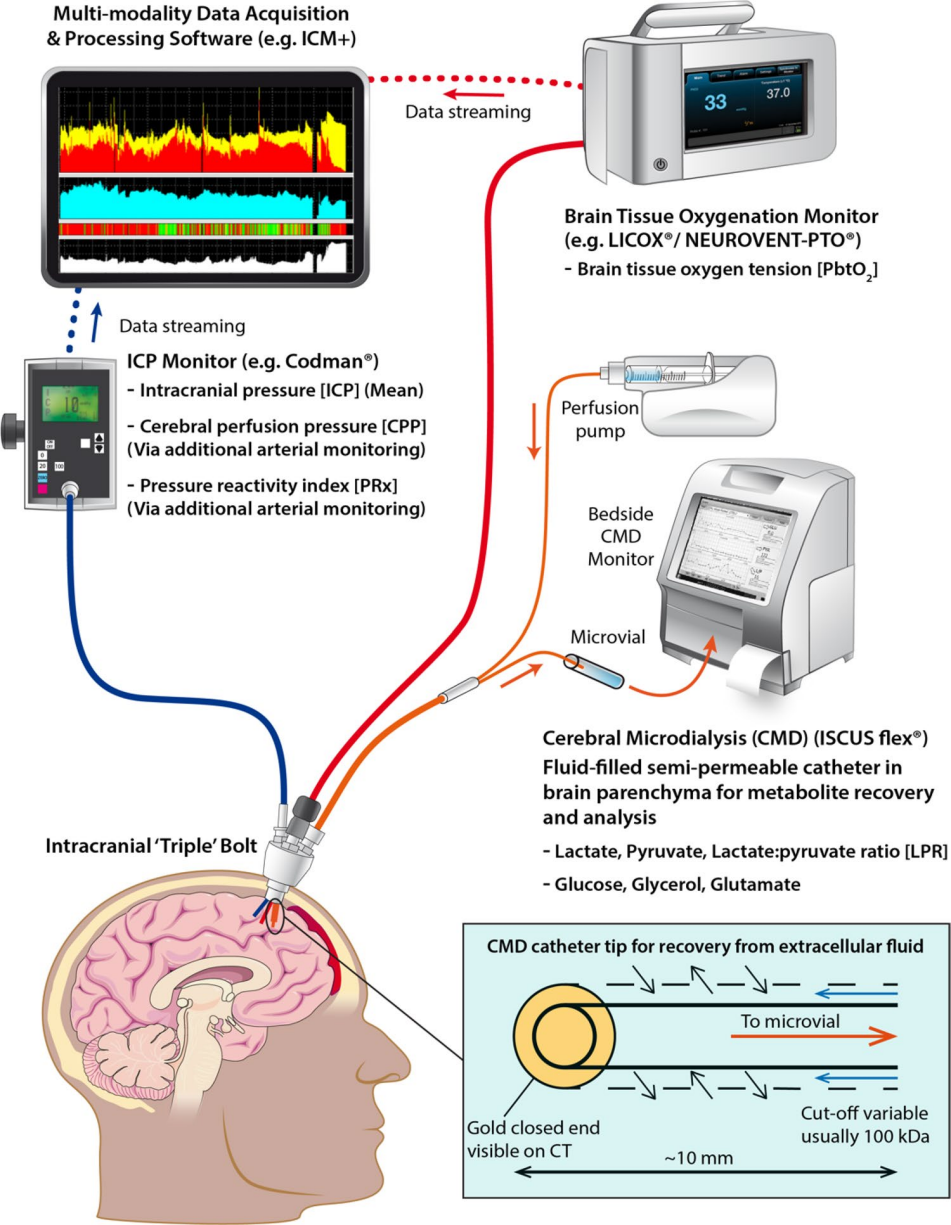
Multi-modality monitor in neurocritical care—illustrating cerebral microdialysis, intracranial pressure and brain tissue oxygenation monitoring. The microdialysis catheter allows sampling of the brain extracellular fluid by recovering molecules of interest that diffuse across the catheter tip and are recovered within a microvial

ICP is the most important goal-directed parameter in the clinical management of severe TBI (Fig. 1). Raised intracranial pressure reduces cerebral perfusion (cerebral perfusion pressure = mean arterial pressure − ICP) risking ischaemia and, when severe and sustained, brain herniation. The Brain Trauma Foundation (BTF) has provided evidence-based guidelines (4th edition, 2016) that summarise the NCCU interventions available for controlling ICP in a staged fashion, with a goal-directed target of 20–25 mmHg [12].

Despite the widespread use of ICP monitoring and acceptance in the TBI community, a recent randomized control trial of ICP monitoring (BEST: TRIP, 2012) was unable to show any benefit [13]. This trial has been severely criticized along two key lines: first, the trial was carried out in units that did not have previous experience of ICP monitoring (to allow ethical clinical equipoise) prior to the trial. Second, both groups of patients had aggressive ICP *therapies* irrespective. Therefore, the trial did not test the utility of ICP interventions but whether the numerical figure from the monitor provided benefit over ‘blind’ management in units with no experience of using the monitors.

### Brain multi-modality monitoring

Brain multi-modality monitoring (MMM) is the use of multiple overlapping monitors to allow early detection of physiological derangements and provide personalised targets for NCCU interventions (Fig. 1). Real-time data acquisition software such as ICM +, CNS Monitor and Bedmaster Ex allow both visualisation and analysis of these parameters at the bedside [14–16]. The definition of pathological targets for these monitors and determining the optimal method of correcting the physiological parameters has underpinned the advances in NCCU treatment of moderate and severe TBI. The two most widely used monitoring probes in addition to intracranial pressure monitors are brain tissue oxygenation and microdialysis monitors.

### Brain tissue oxygenation monitoring

The use of brain tissue oxygen tension (PbtO_2_) monitoring originally arose as a method for avoiding cerebral ischaemia during therapeutic hyperventilation for the control of ICP. The commonest method for monitoring PbtO_2_ is using an invasive probe using a modified Clark electrode, with a typical pathological threshold of 20 mmHg (Fig. 1). In multivariate analysis of outcome, PbtO_2_ has subsequently been shown to impact on outcome. This has led to prospective trials of PbtO_2_ targeted therapy in addition to standard ICP driven care. A phase II trial (BOOST-II, Brain Tissue Oxygen Monitoring and Management in Severe Traumatic Brain Injury) has demonstrated a significant reduction in hypoxia burden (74%) during hospitalization in the PbtO_2_-targeted treatment group with no substantial safety issues. Depending on the study group, directed interventions were used for ICP management (if > 20 mmHg for > 5 min), PbtO_2_ control (if < 20 mmHg for > 5 min) or both [17]. The third phase of the randomized study (BOOST-III) will evaluate the clinical efficacy of “a treatment protocol based on PbtO_2_ monitoring compared to treatment based on ICP monitoring alone” and will enroll patients in the United States [18].

### Cerebral microdialysis (CMD)

Cerebral microdialysis is an invasive monitor that allows sampling of the brain extracellular fluid for cerebral metabolites through a semi-permeable blind-ended intraparenchymal catheter (Fig. 1). It allows for direct measurement and trend profiling of several analytes of which the most important are glucose, lactate, and pyruvate [allowing calculation of the lactate pyruvate ratio (LPR)] typically at hourly intervals [19]. The Consensus Statement from the 2014 International Microdialysis Forum, which thoroughly reviewed the literature on CMD in TBI, recommends a tiered clinical approach to CMD analytes [20]. This Consensus Statement identified LP ratio > 25 and low brain glucose < 0.8 mmol/L as pathological thresholds associated with unfavourable outcomes and necessitating intervention [20–25].

**Fig. 2:**
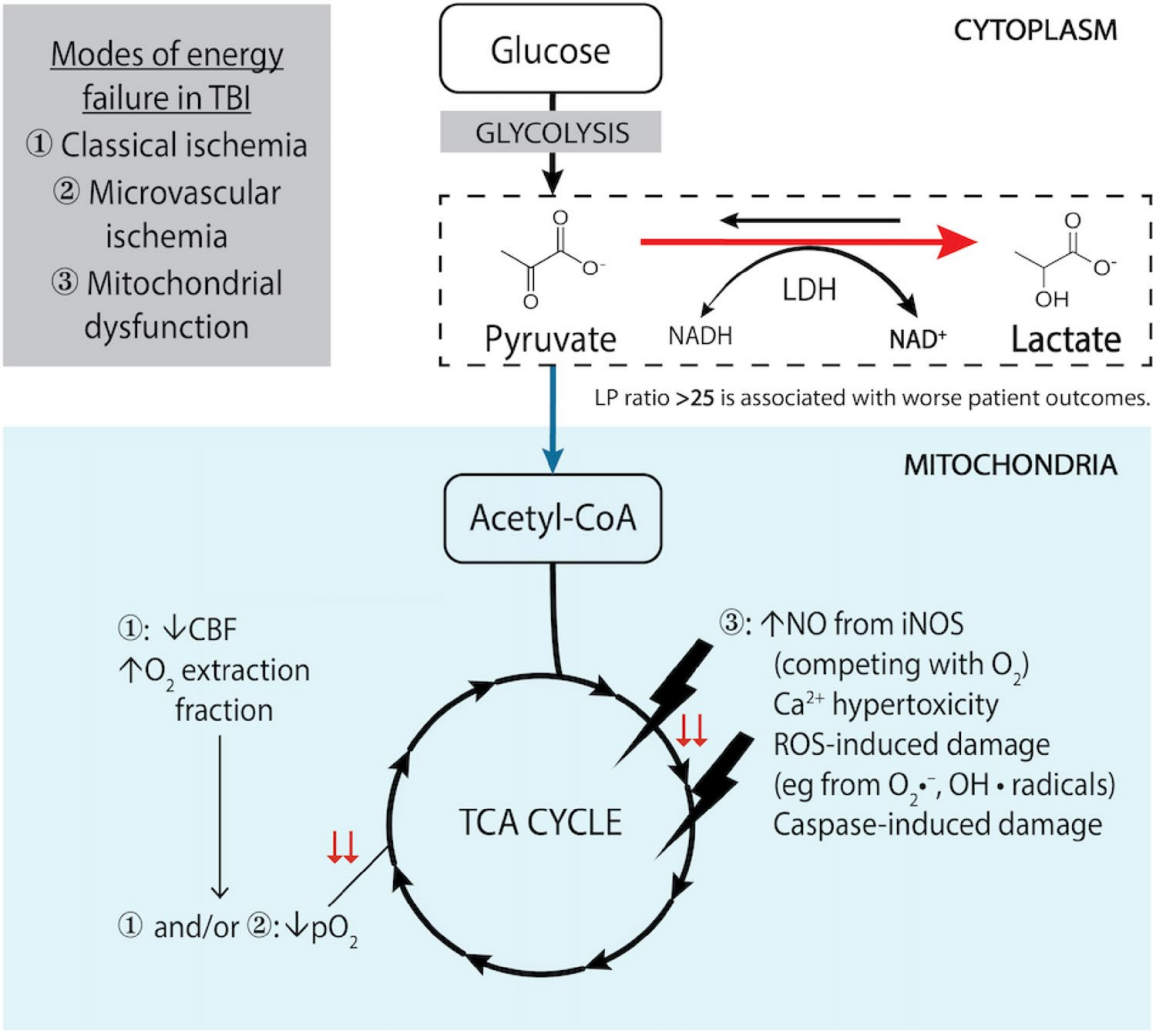
Summary of the mechanisms of energy failure in traumatic brain injury that lead to increased brain lactate: pyruvate ratio (LP ratio). The conversion of lactate to pyruvate is an oxygen-independent step, whereas oxidative phosphorylation and the tricarboxylic acid cycle are oxygen-dependent. Of note, reduced cerebral blood flow and increased oxygen extraction fraction, which characterize classical ischemia, are typically not seen in microvascular ischemia. Mitochondrial dysfunction in the TBI context can arise from multiple pathological processes, often concurrently (most common shown). *Ca*^*2+*^ ionized calcium, *CBF* cerebral blood flow, *iNOS* inducible nitric oxide synthase, *LDH* lactate dehydrogenase, *NAD*^*+*^ nicotinamide adenine dinucleotide (oxidised form), *NADH* nicotinamide adenine dinucleotide (reduced form), *NO* nitric oxide, *O*_*2*_ oxygen, *O*_*2*_·^−^, superoxide radical, *OH*· hydroxyl radical, *pO*_*2*_ tissue oxygen saturation, *ROS* reactive oxygen species, *TCA* tricarboxylic acid cycle

While these parameters are well recognised as independent predictors of outcome over and above clinical parameters and ICP, there is no clearly defined intervention to correct a deranged LP ratio. This reflects the complexity of the underlying pathophysiology such that raised LP ratio can arise from a diverse range of pathologies including classical ischaemia, cortical spreading depression, mitochondrial dysfunction, microvascular collapse and diffusion limited hypoxia [26] (Fig. 2). Prospective protocols that address these issues sequentially are currently being assessed; however, no universally accepted treatment paradigm exists. Nonetheless, CMD has a key advantage over other monitoring tools as it directly assesses the biochemical derangements that occur following TBI, at the cellular level, providing a sensitive monitor of metabolic dysfunction, even if there are several pathological routes to this derangement.

Overall, there is still low-level evidence supporting association of CMD analyte levels with functional, neurophysiological and tissue outcomes as highlighted by a recent systematic review on the topic [27]. Large prospective studies with a multimodal approach are warranted to better profile normal and pathologic values of CMD analytes, and to evaluate associations with patient and tissue outcomes.

### Additional monitoring tools

Conceptually, cerebral blood flow (CBF) is an attractive metric to target within the NCCU; however, the practicalities of measurement have limited its clinical utility. Thermal diffusion flowmetry (TDF) relies on repeatedly heating or cooling a probe and measuring the time to return to baseline as a measure of the ability of cerebral blood flow to buffer temperature towards baseline core temperature. Several monitors are commercially available; yet, they are all limited to variable baseline levels of quantified CBF and are not truly quantitative, making it difficult to target a pathological threshold.

Near infra-red spectroscopy (NIRS) can provide a metric of oxygenated haemoglobin fraction, analogous to pulse oximetry, but is limited by the depth of penetration of the infra-red photons to superficial brain. The signal can also be contaminated by extracranial tissues (such as the temporalis muscle) making absolute quantification of the signal difficult. These two limitations limit the use of this technology to monitoring of the superficial frontal lobes.

### Seizure prophylaxis

Continuous electroencephalography (EEG) is routinely used to monitor patients presenting with post-traumatic seizures (PTS), at increased risk of subclinical seizures or those who are pharmacologically paralyzed. The Brain Trauma Foundation Guidelines suggest that in severe TBI, the rate of clinical PTS may be as high as 12%, while that of subclinical seizures may be as high as 20–25% [28]. There is additional interest in detecting cortical spreading depression as an additional injurious mechanism; however this is reliant on electrodes applied surgically directly to the cortical surface [5]. This has limited the ability to detect CSD systematically.

The BTF Guidelines reinstated the role of post-traumatic seizure prophylaxis with either phenytoin or levetiracetam within 7 days of injury (level IIA recommendation), with particular attention to adverse drug reactions. Benefits from PTS prophylaxis are both acute (limitation of neurophysiological derangements and prevention of herniation) and chronic (prevention of chronic epilepsy for which TBI patients are at higher risk) [28]. A meta-analysis from 2012 showed no significant difference in post-traumatic seizure rates when comparing treatment with phenytoin and levetiracetam in pooled studies [29]. Since then, another large prospective RCT found no difference in rates of early post-traumatic seizures between the patient group treated with phenytoin and the other, with leviteracetam [30]. Long-term disadvantages from anti-convulsant use on neuropsychological recovery in relation with timing and dosage are not yet fully understood and merit further prospective evaluation [28, 31].

### Cerebral perfusion pressure and cerebrovascular autoregulation

The most recent BTF Guidelines recommend a universal CPP target of 60–70 mmHg in severe TBI patients requiring ICP monitoring [28]. There has been a wide interest in how to personalise this target and targeting the endogenous autoregulatory range of an individual patient is conceptually appealing in that the cerebrovascular tree can regulate the downstream neural demands.

**Fig. 3:**
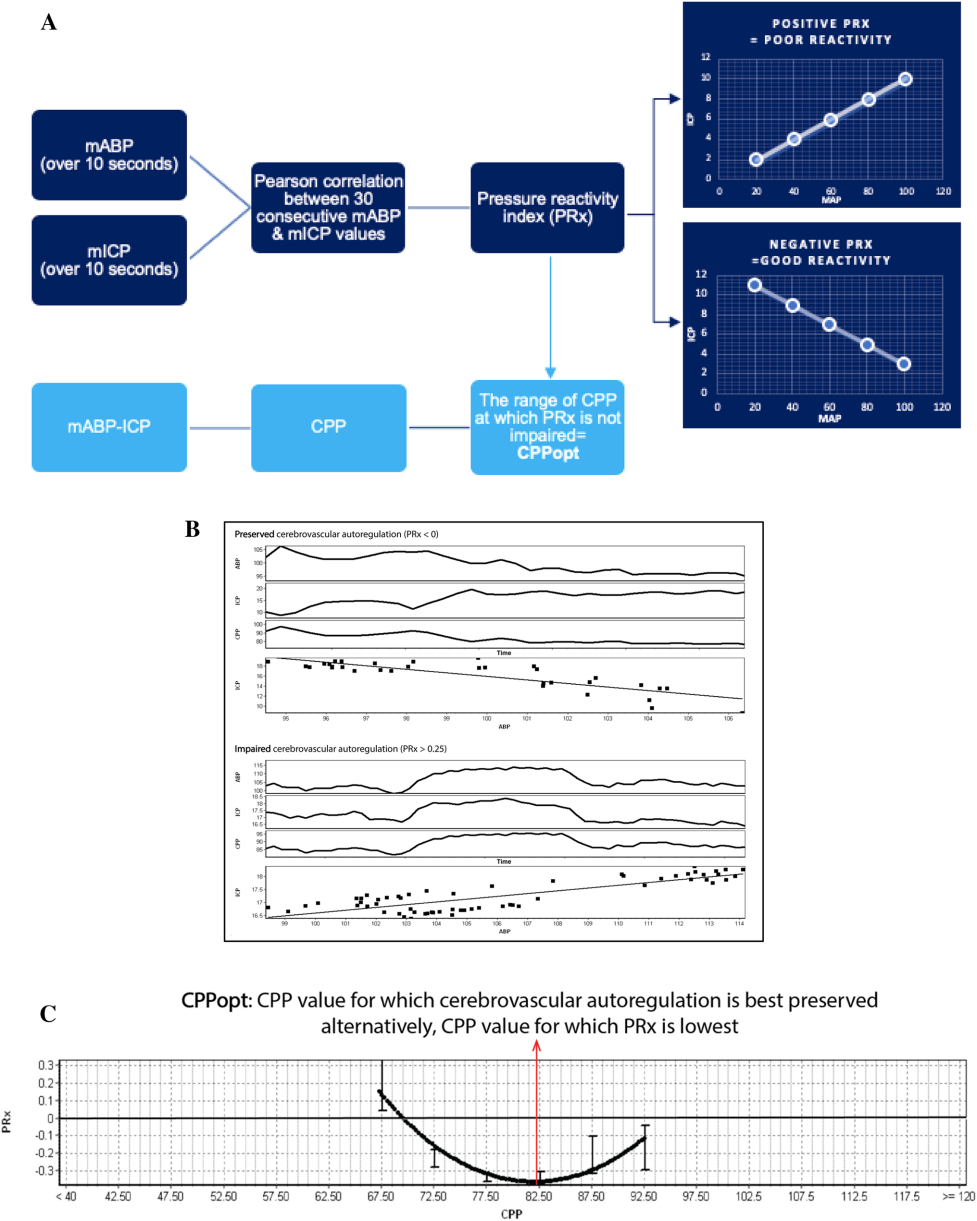
Bar chart of RESCUE-ICP trial outcomes at 6 months [47]. Outcomes are displayed using the Extended Glasgow Outcome Scale (eGOS) on the horizontal axis. eGOS at 6 months represents the primary outcome measure of this trial. The percentage of patients falling within the respective outcome category is displayed in the figure table and illustrated in the corresponding graph. “Favourable” outcomes were defined as upper severe disability or better in the RESCUE-ICP trial. “Unfavourable” outcomes comprise of lower severe disability and vegetative state

Cerebral autoregulation can be assessed using Pulse Reactivity index (PRx), the moving correlation coefficient between mean arterial pressure (MAP) and ICP (Fig. 3). The normal autoregulatory response to increased MAP is vasoconstriction to maintain constant cerebral blood flow, a reduction in arterial blood volume and, therefore, a reduction in ICP. Values above 0.25, whereby increases in MAP lead to an increased ICP, are indicative of impaired autoregulation and correlate with mortality [32–34]. Conversely, negative values, whereby ICP does not increase with increasing MAP, represent vasomotor reaction and prognosticate improved outcomes [35].

Steiner et al. in 2002 introduced the concept of CPPopt which represents the CPP at which PRx is minimised and the cerebral vasculature autoregulates most efficiently (Fig. 3). The CPPopt Guided Therapy Assessment of Target Effectiveness (COGiTATE) study is an ongoing multi-center, phase II non-blinded RCT evaluating the safety and feasibility of maintaining CPP at an individualized target in severe TBI patients recruited < 24 h from injury. This will provide important insight on the feasibility of individualized autoregulation-oriented therapy concept in severe TBI patients.

### Biomarkers

Sustained efforts have been made to identify biomarkers of the injury that results from TBI to detect ongoing injury, to stratify the need for monitoring and interventions and to provide prognostic information. Several biological compartments have been assessed including serum, cerebrospinal fluid, cerebral microdialysate from brain extracellular fluid, and brain tissue. Biomarkers are currently not performed routinely outside of clinical research contexts. Noteworthy biomarkers in TBI include glia-related biomarkers (GFAP, S100B), neuron/axon-related biomarkers (neuron-specific enolase [NSE], neurofilament light polypeptide [NFL], ubiquitin carboxy-terminal hydrolase [UCH-L1], tau, amyloid β, αII-Spectrin breakdown products among others) and other inflammation-related biomarkers (high mobility group box protein 1 [HMGB1], various cytokines and autoantibodies) [33, 34]. To date, only S100B is part of a consensus guideline pathway (by the Scandinavian Neurotrauma Committee) for stratification of mild TBI patients at presentation for CT imaging [36]. No guidelines regarding use of biomarkers in severe TBI exist.

Protein biomarkers with a shorter serum half-life (*t*_1/2_), e.g., S100B (*t*_1/2_ ~ 24 h) are likely more useful than proteins with a longer serum half-life, e.g., NSE (*t*_1/2_ ~ 48–72 h). A longer half-life offers a longer post-injury window for the detection of secondary neurological insults in severe TBI [37].

### Therapeutic hypothermia

There are several plausible mechanisms by which hypothermia can mitigate the effects of TBI including reducing ICP, reducing the innate inflammatory response and reducing the cerebral metabolic rate. These need to be balanced against the risks of coagulopathy, immunosuppression, hypotension, pneumonia, renal impairment and decreased catecholamine responsiveness [28, 38]. Two large phase 3 randomized trials have attempted to show the putative benefit of therapeutic hypothermia. The multi-center non-blinded RCT Eurotherm3235 (2015) is the largest trial on hypothermia for patients with intracranial hypertension (> 20 mmHg) after TBI. This study showed that therapeutic moderate hypothermia (32–35 °C) plus standard care to reduce ICP led to slightly increased mortality rates and unfavourable functional outcomes compared to those with standard care alone. Furthermore, an adverse association between hypothermia and worsening multiple organ failure was noted [39, 40]. A Cochrane Review (March 2016) on mild hypothermia in severe brain injury, which included 37 studies with 3110 participants, demonstrated no high-quality evidence that hypothermia reduces mortality and morbidity in patients with severe TBI [41]. Despite this, hypothermia is routinely used in many units with two staged therapeutic targets, 35 °C and 33 °C.

More recently, the multi-center Prophylactic Hypothermia Trial to Lessen Traumatic Brain Injury—Randomized Clinical Trial (POLAR-RCT, 2018) evaluated outcomes of early, prophylactic, sustained hypothermia (33–35 °C), for at minimum 72 h and up to 7 days, in severe TBI patients. The intention-to-treat population included a total of 500 patients with severe TBI randomized to either normothermia (*n* = 240) or early hypothermia (*n* = 260). The study did not show any benefit from early prophylactic hypothermia in neurological outcomes and mortality at 6 months when compared to normothermia. Intention-to-treat analysis demonstrated increased rates of pneumonia in the hypothermia group (55.0%) vs the normothermia group (51.3%) [42]. This study has been criticized for the use of severe hypothermia in patients prophylactically, even in those without raised ICP. In this group, the risks of hypothermia may exceed the putative benefits.

### Future directions

The introduction of protocolized therapy for moderate–severe TBI has undoubtedly improved outcomes for patients and provided a more consistent management. Nevertheless, the inability to demonstrate a benefit in randomized control trials for established interventions such as ICP monitoring and hypothermia is problematic for the field. When designing trials there is a balance between choosing specific subsets of patients who may benefit from an intervention against the need to recruit sufficient numbers of patients to pragmatically deliver a suitably powered trial. Certainly, the current evidence highlights that universally applying an intervention such as hypothermia without appropriate stratification risks causing harm as much as any benefit.

## Decompressive craniectomy

**Fig. 4:**
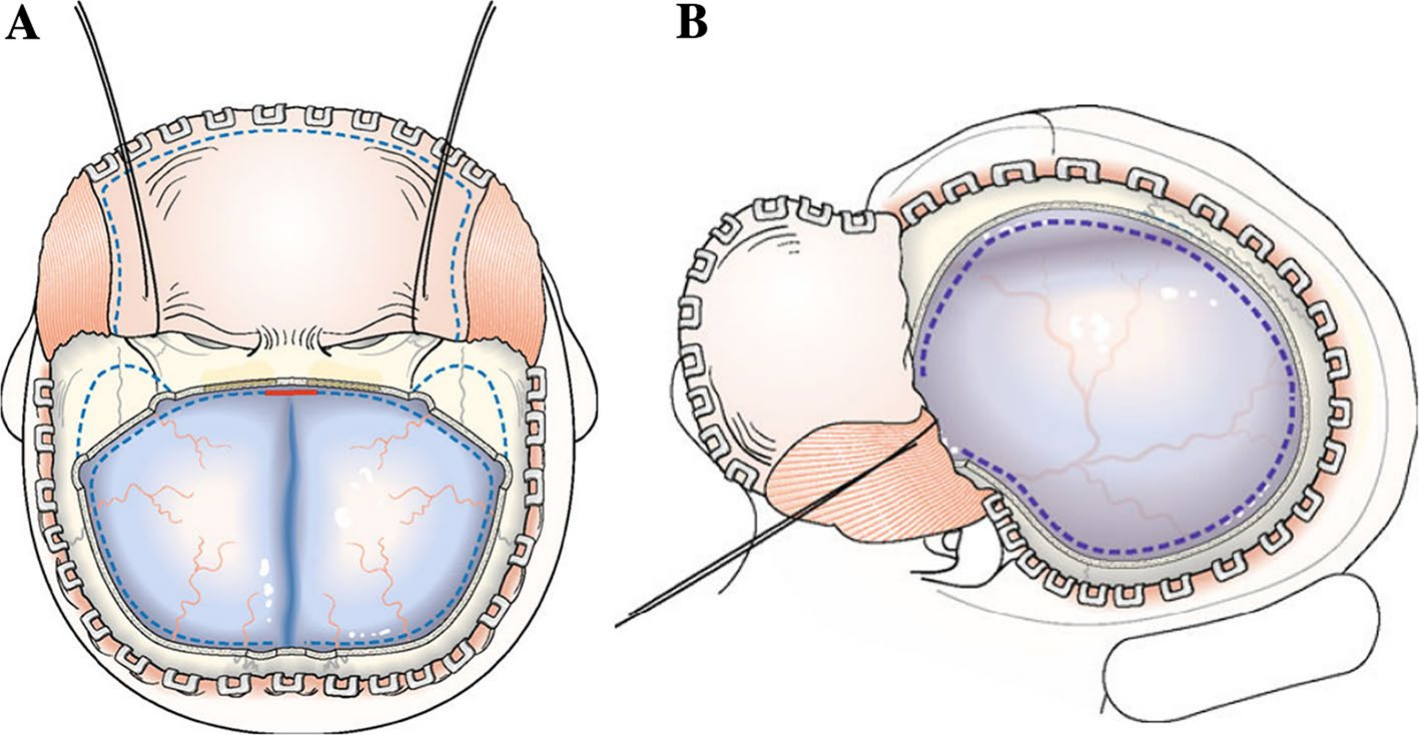
**a** Bifrontal decompressive craniectomy with the dotted line on the dura representing durotomy site and the red line illustrating an area of falxotomy. **b** Decompressive hemi-craniectomy with the dotted line representing durotomy incision. Adapted with permission from Timofeev et al. (2012) [43]

Decompressive craniectomy (DC) is a method of removing a substantial portion of the skull vault to reduce ICP and reduce the consequent deleterious sequelae (Fig. 4) [43]. RCT-based recommendations of trauma DC flap size in refractory raised ICP due to severe TBI suggest the use of 12 × 15-cm flaps is associated with lower mortality (26% vs 35%) and higher Extended Glasgow Outcome Scale (GOS-E) scores when compared to smaller flap sizes [44]. DC can be classified as primary—after evacuation of a haematoma during the acute TBI phase and secondary—independently of haematoma evacuation for ICP control [43, 45].

The use of secondary DC in refractory elevated ICP after severe TBI was explored in the DECRA trial (recruitment 2002–2010), which offered prophylactic DC (within 72 h of TBI) in cases of diffuse (on CT) or severe (on GCS scoring) TBI [46]. 155 cases were randomized to surgical and control arms, where ICP was raised ≥ 20 mmHg for > 15 min within an hour period—despite first-tier medical treatment (i.e., excluding hypothermia and barbiturates use). Importantly, patients who had intracranial haematoma evacuation without primary DC, patients who had a hemi-DC were excluded and patients with bilaterally unreactive pupils were included (forming 27% of surgical arm patients vs 12% of the control group). Mortality rates at 6 months were similar at 19% in the surgery group and 18% medical group. Disability at 6 months in terms of RESCUE-ICP defined (see below) “unfavourable” outcomes were 37% in the surgical group vs 23% in the medical arm, whereas “favourable” were 44% in the surgical arm compared to 59% in the control cohort. After post hoc adjustment for baseline pupil reactivity, functional differences between the two arms were found to be no longer statistically significant [46]. Similar to the POLAR and Eurotherm3235 studies, it appears that unstratified prophylactic use of this intervention does not confer any benefit to patients.

**Fig. 5:**
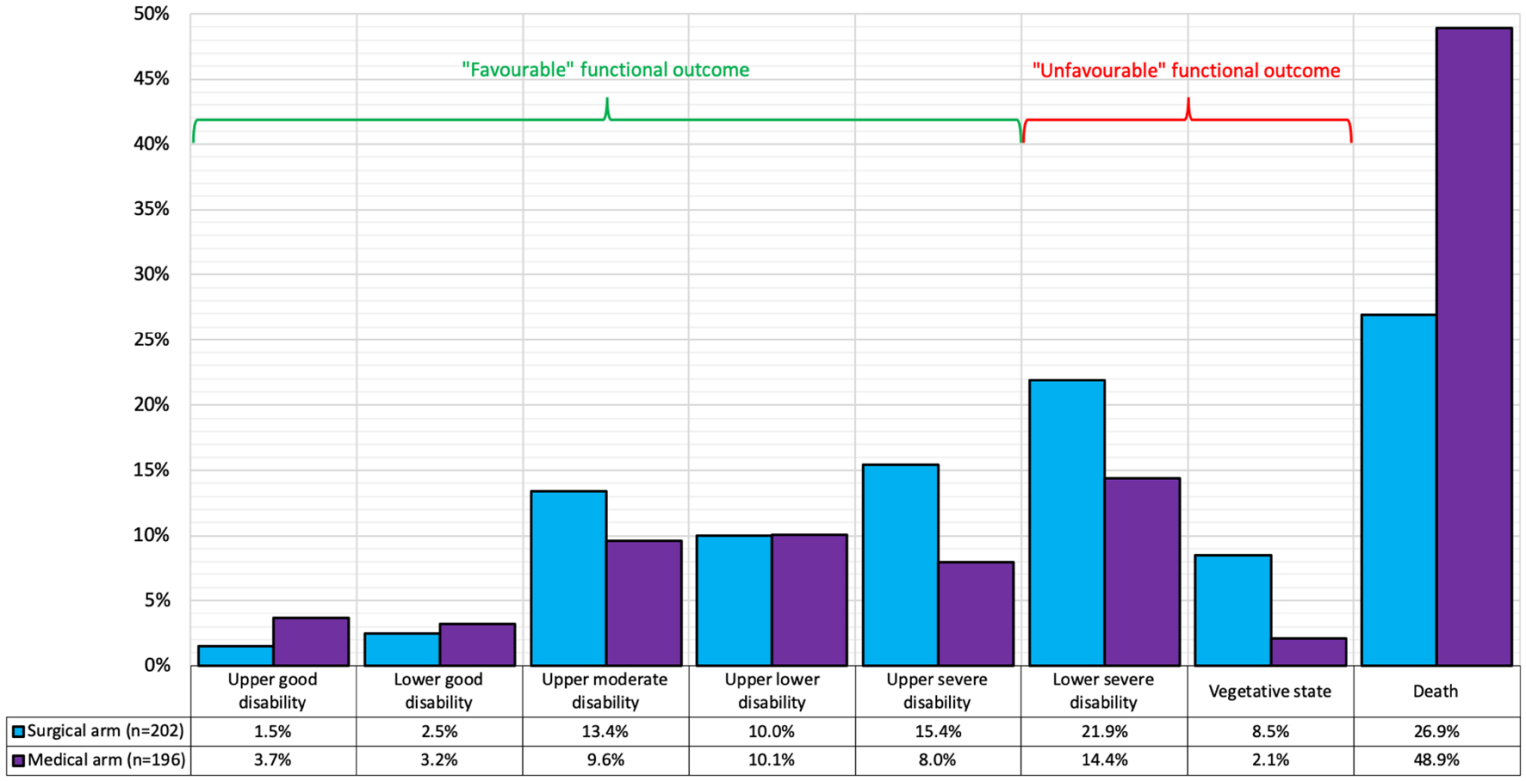
Bar chart of RESCUE-ICP trial outcomes at 6 months (47). Outcomes are displayed using the Extended Glasgow Outcome Scale (eGOS) on the horizontal axis. eGOS at 6 months represents the primary outcome measure of this trial. The percentage of patients falling within the respective outcome category is displayed in the figure table and illustrated in the corresponding graph. “Favourable” outcomes were defined as upper severe disability or better in the RESCUE-ICP trial. “Unfavourable” outcomes comprise of lower severe disability and vegetative state

RESCUE-ICP (recruitment 2004–2014) was an international prospective RCT comparing DC (bifrontal or large unilateral) plus medical management with medical management alone as therapy for TBI patients with severe, sustained and refractory intracranial hypertension [47]. 408 patients were included and randomized when all medical management, other than barbiturate coma, were exhausted and ICP remained elevated > 25 mmHg for 1–12 h. Importantly, patients with fixed bilateral pupils, un-survivable injury, bleeding diathesis or those treated with primary DC were excluded. At 6 months after randomization, secondary DC resulted in lower mortality rates (26.9% vs 48.9% in the medical group). Functional outcomes at 6 months were measured via GOS-E [48] (Fig. 5). Outcomes graded as *upper severe disability* or better were categorised as “favourable” and were seen in 42.8% of surgical cases vs 34.6% of medical cases. “Unfavourable” outcomes (*lower severe disability and vegetative state*) were 30.4% of surgical cases vs 16.5% of medical cases (47). Of note, incidence of persistent vegetative state was 8.5% in the surgical group compared to 2.1% in the medical group. RESCUE-ICP demonstrated that as last tier therapy for raised ICP, DC can reduce mortality, though at a cost of increased severe disability and persistent vegetative state [46, 47, 49]. There is no simple answer as to when DC should be used in severe TBI with medically refractory ICP; however, RESCUE-ICP provided some clear data on the expected outcomes in this context informing the, often complex, discussions with family when this intervention is considered. This study was published after release of the 4th edition of the BTF guidelines.

There is relative paucity in data regarding the use primary DC in TBI, which is more frequently implemented than secondary DC [49, 50]. The currently recruiting RESCUE-ASDH trial is randomizing TBI patients undergoing evacuation of acute subdural haematomas (ASDH) to craniotomy vs with primary DC [49, 51].

### Future directions

One consequence of DC that is undergoing increasing interest, is the need for cranioplasty (replacement of the bony defect with an artificial plate). There is no consensus on the timing of cranioplasty, the cranioplasty material (e.g., titanium, polyethylene, methyl methacrylate, and hydroxyapatite) or the use of autologous bone [49, 52]. It is hoped that initiatives such as the NIHR Global Health Research Group on Neurotrauma and the Collaborative European NeuroTrauma Effectiveness Research in Traumatic Brain Injury (CENTER-TBI) shed light on these questions across resource settings [53, 54].

## Pharmacological therapies for TBI

Despite several decades of successful pre-clinical studies that have developed promising neuroprotective therapies, none have translated into the clinical arena. Unfortunately, there is still no proven curative pharmacotherapy for moderate-to-severe TBI nor pharmacotherapy with unequivocal benefit in functional outcomes. This universal failure has highlighted the biological differences between human and rodent TBI, the lack of investment into mechanistic human TBI studies, the need to accurately define the population who may benefit and the importance of pharmacokinetic studies in humans.

### Corticosteroids

The first large scale pharmacological trial in TBI was the MRC-funded (Medical Research Council, United Kingdom) Corticosteroid Randomisation after Significant Head Injury study (MRC CRASH, 2004), which randomized moderate-to-severe TBI patients to a 48-h infusion of high dose corticosteroids (methylprednisolone) or placebo. 10,008 patients were recruited from 1999–2004 from 239 hospitals in 49 countries. The trial primary outcome was 2-week mortality, which was higher in the treatment group (21.1%) than in the placebo group (17.9%). At 6 months of follow-up, there were 173 excess deaths in the treatment arm (1248 vs 1075). The known complications of corticosteroids such as hyperglycaemia and immunosuppression were not systematically treated in this study [55].

### Progesterone

With the failure of corticosteroids in TBI, pre-clinical animal studies focussed on the early administration of progesterone, a potent neurosteroid synthesized in the central nervous system, and showed that it reduced neuronal loss, cerebral oedema, and behavioral deficits after experimental TBI. Progesterone has then been investigated by large double-blind placebo-controlled -phase III multi-center RCTs (SYNAPSE and PROTECT III published in 2014) without demonstrating benefit in patient mortality and functional outcomes, halting enthusiasm generated by two precedent single-center clinical studies [56–59].

### Erythropoietin

Erythropoietin (EPO) is a glycoprotein regulating haematopoiesis in the bone marrow which is naturally produced in the kidneys following hypoxic stimulation. Animal studies have demonstrated that EPO can neutralize the neuronal apoptotic program, reduce the inflammatory response and act as a neurotrophic factor, thus it is hypothesized to alleviate the impact of secondary brain injury in TBI [60]. EPO use in severe TBI has been controversial owing to select studies showing functional outcome benefits and others finding no significant outcome difference [61, 62]. A meta-analysis of 6 RCTs with 1041 patients (up to January 2017) looking at outcomes of EPO-treated patients vs untreated patients following acute (moderate-to-severe) TBI showed that EPO significantly reduced mortality but did not reduce rates of poor functional outcome. There were no significant differences in complication rates including deep venous thrombosis between the treatment groups [63]. Further well-designed prospective work is required to clarify these findings and determine optimum dosage and treatment timing.

### Amantadine

Amantadine hydrochloride works as a *N*-methyl-d-aspartate (NMDA) antagonist and indirect dopamine agonist [64]. A prospective multi-center double-blind RCT examined amantadine administration vs placebo over 4 weeks followed by 2 weeks in which treatment was discontinued in 184 adult patients who were in a vegetative or minimally conscious state 4–16 weeks after TBI and who were receiving inpatient rehabilitation. The study concluded that amantadine accelerated the pace of functional recovery as measured by the Disability Rating Scale (DRS) during the active 4-week treatment without significant difference in the incidence of serious adverse events in patients with post-traumatic disorders of consciousness [65]. Another small single-center double-blind RCT evaluating amantadine vs placebo given for 6 weeks in 40 severe TBI patients did not show any significant effect on patient mortality and functional outcomes at 6 months [66]. A multi-center double-blind RCT evaluated the efficacy of amantadine vs placebo administered for 60 days on cognitive function in 119 patients with chronic TBI (> 6-month post-injury). This study failed to show benefits from amantadine administration in chronic TBI and even suggested that amantadine may hinder cognitive processing within first 28 days of use [67].

### Tranexamic acid

Tranexamic acid (TXA), a synthetic derivative of the amino acid lysine, is an antifibrinolytic agent used to reduce active bleeding [68]. It works by reversible blockade of the lysine sites on plasminogen [69]. In about 30% of TBI patients, laboratory markers for coagulation are deranged [70]. Furthermore, peri-haemorrhage damage and oedema within the brain is associated with worse neurological outcomes [71].

In the trauma setting (excluding intracranial bleeds), TXA administration has been associated with decreased mortality particularly if used early, elucidated by the CRASH-2 trial [72]. This trial randomized 20,211 adult trauma patients with, or at risk of, significant bleeding randomized within 8 h of injury to either TXA or matching placebo [72]. Subsequently, TXA’s cost-effectiveness in trauma protocols has earned it a spot in the World Health Organization (WHO) List of Essential Medicines [68].

A nested study within CRASH-2 examining the subgroup of trauma patients with extracranial bleeding and TBI with abnormal CT brain findings found a non-significant trend towards reduction in hemorrhage growth, ischaemic lesions, and mortality with TXA administration [73].

These findings have formed the foundation for the Tranexamic Acid for Significant Traumatic Brain Injury (CRASH-3) trial, a RCT aimed at evaluating whether the benefits of TXA are transferable to TBI patients with traumatic intracranial bleeding [68], currently ongoing and eagerly anticipated.

### Citicoline

Citicoline is a cholinergic agent which can increase formation of ATP—hypothesized to promote the functioning of cell-membrane ATP-dependent pumps and thus increase cell-membrane integrity and decrease cellular oedema [74]. In this way, it was considered a potential therapeutic agent against parts of secondary brain injury process. The multi-center double-blind randomized phase III Citicoline Brain Injury Treatment Trial (COBRIT) though failed to demonstrate improvement in functional and cognitive status at 90-day post-injury in moderate-to-severe and mild complicated TBI patients from the use of citicoline vs placebo [75].

### Anti-inflammatory therapies

Recombinant interleukin-1 receptor antagonist (rIL1ra) has demonstrated putative benefit in a range of neuronal pathologies by inhibition of the IL1 receptor mediated inflammatory cascade [76, 77]. In TBI it has been shown to be safe and modify the acute neuroinflammatory response in a phase II single-center RCT [78]. A dose ranging study to optimise both the dose and timing of administration is currently in progress [79].

## Conclusions

TBI is a major global health challenge and priority. There are several promising lines of research directed towards optimising neurointensive care protocols, developing an evidence base for surgical intervention, and translating promising neuroprotective pharmacotherapies into application to human pathology. The importance of personalised medicine and combinatorial therapy in the field of TBI cannot be overstated given the heterogeneity of the pathology such that a single intervention cannot address every pathological mechanism at play. This is at odds with conventional randomized control trial methodology and may underpin the large number of failures of RCTs to demonstrate any benefit in this pathology.
